# Pulmonary flow-study can predict in-hospital prognosis of unifocalization and corrective repair of pulmonary atresia/ventricular septal defect with major aortopulmonary collateral arteries

**DOI:** 10.1016/j.heliyon.2024.e27109

**Published:** 2024-02-25

**Authors:** Xiaofeng Wang, Zhiyuan Zhu, Zhongyuan Lu, Wenlong Wang, Xu Wang

**Affiliations:** Department of Pediatric Intensive Care Unit, National Center for Cardiovascular Disease, Fuwai Hospital, Chinese Academy of Medical Sciences and Peking Union Medical College, Beijing, 100037, People's Republic of China

**Keywords:** Pulmonary atresia, Major aortopulmonary collateral arteries, Unifocalization, Corrective repair, Pulmonary flow study

## Abstract

**Objectives:**

With the development of perioperative treatment, the results of the unifocalization and corrective repair of pulmonary atresia/ventricular septal defect with major aortopulmonary collateral arteries have been significantly improved. However, the in-hospital recovery is quite different individually. Therefore, it is essential to find prognostic indicators to avoid unsatisfactory recovery.

**Design:**

This was a case-control study.

**Setting:**

The study was conducted in the national center for cardiovascular diseases in China.

**Participants:**

Pediatric patients between 2014 and 2022.

**Interventions:**

None.

**Measurements & main results:**

A total of 19 patients were included. The possible prognostic indicators included were commonly used clinical data. Unsatisfactory postoperative recovery was defined as mechanical ventilation≥ 7 days and/or in-hospital death. Satisfactory postoperative recovery was defined as mechanical ventilation<7 days and survival at discharge. We separated patients into two groups and compared the peri-operative data through univariable analysis. There were 8 patients in unsatisfactory recovery group (including 1 death) and 11 patients in satisfactory recovery group. Among all the possible prognostic indicators, through univariable analysis, pulmonary arterial pressure in pulmonary flow study was statistically different (p = 0.027 < 0.05). The ROC curve showed that the area under curve and cut-off values in predicting satisfactory recovery were 0.841 and 22 mmHg; the corresponding sensitivity and specificity were 100% and 72.7%. There was no statistical difference between the two groups in ventricular septal fenestration and pulmonary hypertension targeting drugs.

**Conclusion:**

A pulmonary arterial pressure <22 mmHg in pulmonary flow study may avoid unsatisfactory in-hospital recovery after unifocalization and corrective repair of pulmonary atresia/ventricular septal defect with major aortopulmonary collateral arteries.

## Abbreviations

PA =: pulmonary atresia
VSD =: ventricular septal defect
MAPCAs =: major aortopulmonary collateral arteries
TNPAI =: total neo-pulmonary artery index
RVSP =: right ventricle systolic pressure
SBP =: systolic blood pressure

## 1 Introduction

Pulmonary atresia with ventricular septal defect (PA/VSD) is a complex congenital heart anomaly. It is reported in 4–10 infants out of 100 000 live births, accounting for about 2% of children with congenital heart disease [1,2]. PA/VSD varies from relatively simple to complex. In complex cases, the main pulmonary artery or its main branch is atresia, and part or all of the pulmonary parenchyma is supplied by major aortopulmonary collateral arteries (MAPCAs) arising from the aorta or its main branch.

The main goal of surgical treatment for complex form of PA/VSD and MAPCAs is to ensure adequate pulmonary blood flow without over circulation, especifically to:1) establish a confluent, functional pulmonary vasculature, 2) set up a right ventricular to pulmonary arterial connection and 3) repair the VSD with a patch. The unifocalization procedure for MAPCAs is a means of incorporating all available lung segments so that the VSD can be closed without significant right ventricular hypertension. However, improper decision for VSD closure may be associated with high mortality and morbidity rate [3].

It is important to assess the adequacy of pulmonary vasculature and right ventricle afterload, by using schemes for objectively measuring, such as total neo-pulmonary artery index (TNPAI) [4] and the ratio of right ventricle systolic pressure and systolic blood pressure (RVSP: SBP) [5]. The pre-operative TNPAI≥150 mm^2^/m^2^ and post-operative RVSP: SBP≤0.7–0.8 were considered as indicators to close VSD. In addition, due to the sub-optimal predictability of TAPNI and the salvage measure of the RVSP: SBP ratio, decisions are sometimes based on intra-operative assessment of pulmonary blood flow (estimated mean pulmonary arterial pressure) with the growing number of studies coming to a consistent conclusion in recent years. The estimated mean pulmonary arterial pressure ≤25–30 mmHg was considered as a more reliable indicator when closing the VSD [6].

Although the medium-term postoperative mortality is acceptable following all the indications, they are still imperfect, and the in-hospital postoperative recovery varies widely during clinical treatment. Even if the patients met all the current indications, part of them still experienced unsatisfactory recovery, such as unstable postoperative hemodynamics, multiple organ dysfunction, delayed postoperative recovery and even death. In addition, although attention has been paid to the pulmonary flow study in recent years, the use of target drugs with the purpose of improving the postoperative in-hospital prognosis relies more on experience rather than evidence. In patients with borderline pulmonary vascular development (similar to grey zone in pulmonary hypertension), it has also been reported that repairing VSD with fenestrated patch (sub-complete repair) shows satisfactory mid or long-term follow-up results, which made it a promising corrective surgical method. However, there was no study specifically describing the indicator of satisfactory in-hospital postoperative recovery of these patients. Therefore, this study is aimed to find prognostic indicators to avoid unsatisfactory in-hospital recovery of this complicated cardiac procedure. The primary outcome of the study is to prove the utility of pulmonary flow-study to predict in-hospital prognosis. The secondary outcome is to find other possible indicators which can improve in-hospital prognosis.

In distinguishing satisfactory or unsatisfactory in-hospital recovery, in addition to mortality rate, we choose prolonged mechanical ventilation (≥7 days) as the criterion [7], as the duration of mechanical ventilation is a management variable after congenital heart disease surgery.

## 2 Patients and methods

### 2.1 Patients

We retrospectively studied patients from January 2014 to August 2022 in Fuwai hospital. Patients were identified through the cardiac database, and the medical records were subsequently reviewed. The inclusion criteria: 1) All patients who were diagnosed with PA/VSD and MAPCAs using echocardiography and angiography. 2) Patients who underwent one-stage or staged unifocalization and VSD close procedure with or without fenestration. The exclusion criteria: 1) Patients with PA/VSD and MAPCAs related to double-outlet right ventricle, congenital corrected transposition of the great arteries or single-ventricle physiology. 2) Patients who got only traditional assessment but did not get pulmonary flow study assessment. All of the patients underwent repair by the same surgeon.

### 2.2 Surgical strategy

In patients with PA/VSD and MAPCAS, among all traditional pre-operative assessment (physical examination, electrocardiogram, X-ray, echocardiogram, angiography), the decisions made for corrective repair requirements were mainly depended on the predicted TNPAI≥150 mm^2^/m^2^ after unifocalization. According to the development of pulmonary vascular bed, part of the patients needed palliative surgery (modified Blalock-Taussig shunt or right ventricle-pulmonary artery connect) before corrective repair in our center. In those patients, at least 3–6 months follow-up time was performed for the final operative decision.

The pulmonary flow study was not a conventional intraoperative evaluation. It was not conducted for patients whose pre-operative assessments showed that corrective repair was obviously suitable, or palliative surgery was obviously suitable. We followed the relevant technique process of the pulmonary flow study reported by Stanford University. A pulmonary arterial pressure ≤25–30 mmHg measured by pulmonary flow study was considered as indication for corrective repair. In some selected patients, although the pulmonary arterial pressure was more than 30 mmHg, we also closed the VSD with fenestration after comprehensively evaluated other indications (such as SpO_2_ or TNPAI) in early stage. Through our learning curve and related research, the criterion for closing VSD was no more than 25 mmHg.

Surgical technique involved right ventricular outflow tract reconstruction, unifocalization of MAPCAs, and VSD closure via median sternotomy. The right ventricular outflow tract reconstruction was performed with hand-made valved GORE-TEX conduit, or conduit with bioprosthetic valve, or homograft conduit. MAPCAs were dissected without cardiopulmonary bypass. Unifocalization was done on the beating heart under cardiopulmonary bypass. We unifocalized all the MAPCAs that were not in communication with the native pulmonary arteries and ligated or occluded the MAPCAs that were also supplied to the lung segments by the native pulmonary arteries. For patients with distal pulmonary stenosis, pulmonary angioplasty was performed. In our center, patients with increased pulmonary artery pressure >25 mmHg measured by pulmonary flow study, we performed VSD fenestration to avoid rebuilding extracorporeal circulation for secondary VSD fenestration or reopening due to high right ventricle pressure.

### 2.3 Postoperative therapy

Sedative and analgesic treatment with midazolam and fentanyl, respectively, were initiated when patients were admitted to intensive care unit. The prescription of muscle relaxants (pancuronium bromide or cisatracurium besilate) by continuous infusion or bolus infusion was based on the hemodynamic conditions. The aim of mild hyperventilation was 30–35 mmHg pCO_2_. The aim of alkalemia was to maintain pH at 7.4–7.5. Vasoactive drugs were given, including dopamine (3–8 μg/kg/min), dobutamine(3–8 μg/kg/min), milrinone (0.3–1 μg/kg/min), epinephrine (0.03–0.1 μg/kg/min), and vasopressin (0.005–0.03 μg/kg/h), depending on the hemodynamic status. Continuous intravenous infusion of torasemide (0.1–0.2 mg/kg/h) was prescribed for diuretic treatment.

For patients with acute kidney injury, renal replacement therapy was initiated when urine volume was under 0.5 mL/kg/h for 6–12 h. The types of renal replacement therapy included peritoneal dialysis and continuous veno-venous hemofiltration therapy.

When a patient was transferred from operation room to intensive care unit, the on-duty ICU doctors and surgeons would decide whether to prescribe inhaled nitric oxide therapy. The administration of nitric oxide gas is accomplished by a delivery and monitoring system (SLE3600 INOSYS, UK). The system is connected to the patients via the side branch of the ventilator. In our center, the chosen inhaled nitric oxide concentration is 20 ppm, and the necessity of inhaled nitric oxide therapy is re-evaluated every 24 h. When inhaled nitric oxide therapy was withdrawn, oral sildenafil (0.25 mg/kg/dose, QID) and bosentan (2 mg/kg/dose, BID) were prescribed.

### 2.4 Data collection

Perioperative data were collected retrospectively by review of hospital records and reports of referring cardiologists. Mortality referred to the death after the last procedure performed and defined as within hospital stay. The TNPAI = the sum of cross-sectional area of inherent pulmonary artery and the unifocalized collateral vessels (mm^2^) divided by the body surface area (m^2^). The RVSP were measured by direct needle manometry after weaning from cardiopulmonary bypass in the operation room.

### 2.5 Grouping

Patients were separated into two groups. The group 1 was unsatisfactory in-hospital recovery group and the group 2 was satisfactory in-hospital recovery group. Unsatisfactory in-hospital recovery was defined as patients who met at least one of the criteria: severe complications (e.g. death) and/or delayed recovery (mechanical ventilation≥ 7 days). Those who presented none of the above criteria was defined as satisfactory in-hospital recovery.

### 2.6 Statistical analysis

All statistical analyses were performed by SPSS 25.0 (IBM Corp., Armonk, NY, USA). Continuous variables were expressed as mean ± SDs or as median with interquartile ranges for parametric and nonparametric variables. Classified variables are represented by numbers (percentages). Kolmogorov–Smirnov test was used to determine normality of data. The Student's *t*-test and Wilcoxon rank sum test were used for continuous variables when comparing data between two groups. The chi-squared test was used for classified variables. Binary logistic regression was used to determine the correlation between variables and outcomes. The ROC curve was used to show the sensitivity, specificity, area under the curve, cut off point of variables. A P-value <0.05 indicated statistically significant differences.

## 3 Results

### 3.1 Overall demographic, surgical and postoperative characteristics

There were 19 patients included in our study. In all the 19 patients, 8 of them were male, 11 of them were female, the median age was 27 (8, 44) months, the mean body weight was 11 ± 5 kg. The total number of MAPCAs was 62, with the median number of 3.3 MAPCAs per patient. The pre-operative SpO_2_ was 83 ± 10 %. Two of them presented noncardiac malformation. There were 4 patients with non-confluent pulmonary arteries, 13 patients with pulmonary artery stenosis and 8 patients with palliative surgery history. Among all the 62 MAPCAs, 37 of them were unifocalized (1–5 per patients), and others were occluded or ligated. The TNPAI was 200 ± 51 mm^2^/m^2^. The pulmonary arterial pressure in pulmonary flow study was 22 ± 7 mmHg. The postoperative RVSP: SBP ratio was 0.7 ± 0.2. The cardiopulmonary bypass duration was 236 ± 56 min. The aortic cross-clamp duration was 75 ± 35 min. The median mechanical ventilation duration was 140 (42, 283) hours. The median intensive care unit duration was 8 (5, 17) days and median postoperative length of stay was 19 (13, 38) days. All the VSD fenestration were preventive, and no secondary fenestration or reopening was performed in our study. There were 6 patients who received sequential pulmonary targeted therapy, 5 patients who received renal replacement therapy, 2 patients who received tracheotomy therapy. There was 1 postoperative death in all the 19 patients (mortality rate: 5.3%). The death patient's pulmonary arterial pressure was 35 mmHg in pulmonary flow study, and the preoperative SpO_2_ was 84% and TNPAI was 190, which failed in flow study exam but passed the traditional indications of corrective surgery. Although the VSD fenestration was performed, he still has significant postoperative right ventricular dysfunction and multi-organ insufficiency. After renal replacement therapy, triad targeted drug therapy for pulmonary arterial hypertension and tracheotomy, the patient could not wean from mechanic ventilation and died 55 days after surgery. The cause of death was right ventricular failure.

### 3.2 Grouped data and univariable result

There were 8 patients in unsatisfactory recovery group (group 1) and 11 patients in satisfactory recovery group (group 2). The grouped data of demographic, surgical characteristics and the statistical difference between the two groups were shown in Table 1. Through univariable logistic regressions, the pulmonary arterial pressure in pulmonary flow study was statistically significant, with p = 0.027, exp(B) = 1.366 and the 95% CI: 1.036 to 1.800.

**Table 1:** The grouped demographic and surgical characteristics.

	Group 1 n = 8	Group 2 n = 11	p-value
Age (month)	13 （7, 43）	31 (21, 58)	0.33
Gender			0.551
Male	3	5	
Female	5	6	
Weight (kg)	9.8 ± 4.6	12.1 ± 4.4	0.269
SpO_2_ (%)	85 ± 9	81 ± 10	0.492
Extracardiac disease	2	0	0.164
Palliative surgery history	3	5	0.551
Non-confluent pulmonary arteries	2	2	0.574
Pulmonary artery stenosis	6	7	0.494
Unifocalized MAPCAs	2.4 ± 1.2	1.6 ± 0.7	0.102
TNPAI	209 ± 46	194 ± 56	0.554
Pulmonary pressure in flow study	28 ± 4	19 ± 6	0.003*
RVSP:SBP ratio	0.72 (0.51, 0.9)	0.73 (0.64, 0.79)	1.0
Cardiopulmonary bypass	260 ± 64	218 ± 44	0.102
Aortic cross-clamp	71 ± 32	78 ± 39	0.68
VSD fenestration	4	3	0.297
Targeted therapy	2	6	0.208

MAPCAs = major aortopulmonary collateral arteries, TNPAI = total neo-pulmonary artery index, RVSP = right ventricle systolic pressure, SBP = systolic blood pressure, VSD = ventricular septal defect.

### 3.3 ROC results

**Fig. 1:**
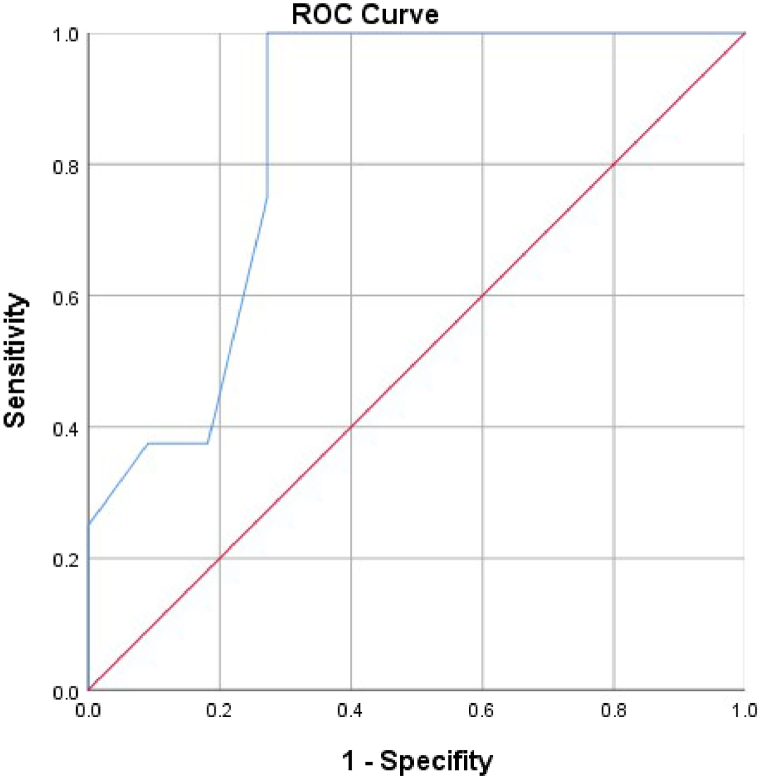
the ROC curve result of pulmonary arterial pressure in pulmonary flow study.

As is shown in the ROC curve, we found that the area under curve of pulmonary arterial pressure in pulmonary flow study was 0.841, cutoff point was 22 mmHg, sensitivity was 1.0, specificity was 0.727. The ROC curve was shown in Fig. 1.

## 4 Discussion

Patients with PA/VSD and MAPCAs remain one of the most challenging cardiac anomalies. The key to surgical treatment is the early rescue of intrinsic pulmonary artery and MAPCAs. As for the treatment of MAPCAs, different centers may have different strategies. In our center, for patients with pulmonary artery dysplasia, the first step is to promote the development of the intrinsic pulmonary arteries by increasing pulmonary blood flow (Blalock-Taussig shunt or right ventricle-pulmonary artery connection) [8]. Ligation or occlusion of collateral arteries is preferred in cases of dual blood supply and adequate development of intrinsic pulmonary vessels. In our center, unifocalization surgery could be avoided unless a certain lung segment only receives the collateral blood supply. For these patients, the key point is that early operation is essential to improve pulmonary vascular development in order to avoid progressive pulmonary artery dysplasia, or to avoid the obstructive pulmonary vascular disease due to the collateral pulmonary vessels. The above two pathophysiological changes can lead to segmental pulmonary hypertension [9].

However, closing VSD is not in emergency. Once corrective surgery is performed for inappropriate patients, the increased pulmonary artery pressure will lead to deteriorating right ventricular afterload, resulting in serious postoperative complications and delayed recovery. Even if the patient can survive in the perioperative period, long-term pulmonary hypertension will inevitably lead to progressive right heart failure and eventual death. Patients with the unfavorable anatomy (hypoplastic MAPCAs) remain a challenge with high mortality rate [10]. Therefore, in the decision of closing the VSD, comprehensive and accurate evaluation should be performed before and during the operation to ensure that postoperative right ventricular pressure is within the safety range [11].

After weaning from cardiopulmonary bypass, patients with increased RVSP can be remedied by salvage VSD fenestration. However, in such complex surgery, right heart dysfunction due to secondary extracorporeal circulation should be avoided, because it is associated with higher postoperative mortality rate [12]. In this study, no difference in RVSP was found between the two groups. We speculated that it may due to the residual pressure gradient of the neo-pulmonary valve, the value of RVSP cannot accurately reflect the degree of pulmonary vascular disease.

The professor Hanley in Stanford university creatively invented the method of intraoperative pulmonary flow study to evaluate the indication of closing the VSD [13], in their study, 28 of all the 35 patients underwent one stage corrective repair based on the results of flow-study, and no postoperative death occurred. With the evolution of pulmonary flow study, the quantity of flow increased from 2.5 L/min to 3 L/min, and the corresponding cutoff point also decreased from 30 mmHg to 25 mmHg, suggesting that the indication for closing VSD has become increasingly strict [14,15]. According to the research by Zhu and his colleagues in Toronto [3], 34 of all the 40 flow-studied patients underwent corrective repair. Six of the 40 patients passed away during 2.2 years of follow-up, the cut-off point of 25 mmHg was associated with better medium- and long-term survival rate. In 2019, based on the research in the coupling of pulmonary artery pressure and right ventricular function, the 6th world symposium on pulmonary hypertension has revised the diagnostic standard decreasing pulmonary hypertension from 25 mmHg to 20 mmHg (mean pulmonary arterial pressure) [16], suggesting that even slightly increased pulmonary artery pressure may have adverse effects on the right heart function, quality of life and survival rate of pulmonary arterial hypertension patients. However, this new standard has not been applied to the pulmonary flow study.

Our center has adopted flow study methods in 2014 when it became a mature technology. But in terms of decision making, there was a learning curve. In the beginning, as the standard of pulmonary arterial hypertension was 25 mmHg (mean pulmonary arterial pressure), we speculated that for patients with slightly increased pulmonary arterial pressure, the VSD fenestration could be performed so that in case of increased pulmonary vascular resistance, the right ventricular afterload could be reduced by right-to-left shunt [17]. Meanwhile, restricted VSD will not cause unacceptable hypoxemia. However, for the subsequent postoperative treatment, we did not find that the fenestration could significantly improve the postoperative recovery process.

In this study, through statistical analysis, we found that only pulmonary arterial pressure in pulmonary flow study was associated with satisfactory in-hospital recovery, suggesting that hemodynamic examination of pulmonary vascular disease has the most obvious clinical significance. Moreover, different from previous literatures, the cut-off value of estimated mean pulmonary arterial pressure for satisfactory postoperative recovery was 22 mmHg, which was between the current diagnostic criterion for pulmonary arterial hypertension (20 mmHg) and the classic standard for corrective repair (25 mmHg). This result suggested that the standard of corrective surgery may be stricter than before. However, due to the small sample size of this study, further research is needed to confirm the cut off value at 22 mmHg as final criterion.

The safety and efficacy of targeted therapy for pulmonary arterial hypertension in this special classification of pulmonary arterial hypertension (segmental pulmonary arterial hypertension) is still controversial [18,19]. Even the classification of this pulmonary vascular disease is not conclusive, the 2020 European Society of Cardiology guidelines for adult congenital heart disease classify it into the first category of pulmonary arterial hypertension based on the pathological findings and therapeutic results from current literature [20]. On the contrary, according the 2022 European Society of Cardiology pulmonary arterial hypertension guidelines, segmental pulmonary arterial hypertension is still in class 5, which means the targeted therapy is not recommended by pulmonary vascular specialist for patients with segmental pulmonary arterial hypertension [21]. In clinical practice, our intensive care unit doctors usually provide targeted therapy for these patients based on experience, because inhaled nitric oxide treatment can be used to stabilize hemodynamics as the most effective targeted therapy. However, recent studies on inhaled nitric oxide treatment has indicated that this drug was proved to be useful only in acute pulmonary vasodilatation test and the rescue of pulmonary hypertension crisis. All researches failed to prove that inhaled nitric oxide can improve post-operative recovery in patients after cardiac surgery [[22], [23], [24]]. In our study, the targeted therapy shows no effort on improving the prognosis after complex cardiac surgery, which is highly consistent with recent researches. This result suggests that targeted therapy may be less helpful than expected in patients with this complex pulmonary vascular disease.

## 5 Research limitation

There are some shortcomings of this study. This is a single center study and has a small sample size, which may ignore the statistical difference of important variables. ROC curve data may also be affected by the sample size. This deficiency can be solved by expanding the sample size in the future. As there is no clear standard for surgical indications to PA/VSD and MAPCAS at present, it is difficult to sign informed consent so that prospective studies cannot be conducted. This study focused on early postoperative recovery, but further studies focused on medium and long-term (at least 1–2 years) survival quality and hemodynamics can be carried out in the future to better evaluate the real utility of flow-study.

## 6 Conclusion

The pulmonary pressure under 22 mmHg in pulmonary flow study may avoid unsatisfactory in-hospital recovery after unifocalization and corrective repair of PA/VSD and MAPCAs.

## Ethical statement

The approval of the Fuwai hospital Ethics Committee was obtained in October 2022, ID: 2022–1859. The ethical principles followed the 1975 Helsinki Declaration. This was a retrospective analysis based on anonymized data collected for routine clinical care and administrative purposes, so individual informed consent was waived.

## Funding statement

This work was supported by the 10.13039/501100018684National Health Commission of the People's Republic of China (grant number 2022-GSP-GG-32).

## Data availability statement

The data associated with may study has not deposited into a publicly available repository, and the data in this article will be shared on reasonable request to the corresponding author.

## CRediT authorship contribution statement

**Xiaofeng Wang:** Writing – review & editing, Writing – original draft, Investigation, Formal analysis, Conceptualization. **Zhiyuan Zhu:** Writing – review & editing, Formal analysis. **Zhongyuan Lu:** Writing – review & editing, Conceptualization. **Wenlong Wang:** Writing – review & editing, Conceptualization. **Xu Wang:** Validation, Supervision, Project administration, Methodology, Funding acquisition.

## Declaration of competing interest

The authors declare the following financial interests/personal relationships which may be considered as potential competing interests:Xu Wang reports article publishing charges was provided by National Health Commission of the People's Republic of China. Xu Wang reports a relationship with National Health Commission of the People's Republic of China that includes: funding grants. If there are other authors, they declare that they have no known competing financial interests or personal relationships that could have appeared to influence the work reported in this paper.
